# Joint High-Order Synchrosqueezing Transform and Multi-Taper Empirical Wavelet Transform for Fault Diagnosis of Wind Turbine Planetary Gearbox under Nonstationary Conditions

**DOI:** 10.3390/s18010150

**Published:** 2018-01-07

**Authors:** Yue Hu, Xiaotong Tu, Fucai Li, Guang Meng

**Affiliations:** State Key Laboratory of Mechanical System and Vibration, Shanghai Jiao Tong University, Shanghai 200240, China; huyue_sjtu@sjtu.edu.cn (Y.H.); tormii@sjtu.edu.cn (X.T.); gmeng@sjtu.edu.cn (G.M.)

**Keywords:** wind turbine gearbox, fault diagnosis, synchrosqueezing transform, multi-taper, empirical wavelet transform, time-frequency analysis

## Abstract

Wind turbines usually operate under nonstationary conditions, such as wide-range speed fluctuation and time-varying load. Its critical component, the planetary gearbox, is prone to malfunction or failure, which leads to downtime and repair costs. Therefore, fault diagnosis and condition monitoring for the planetary gearbox in wind turbines is a vital research topic. Meanwhile, the signals measured by the vibration sensors mounted in the gearbox exhibit time-varying and nonstationary features. In this study, a novel time-frequency method based on high-order synchrosqueezing transform (SST) and multi-taper empirical wavelet transform (MTEWT) is proposed for the wind turbine planetary gearbox under nonstationary conditions. The high-order SST uses accurate instantaneous frequency approximations to obtain a sharper time-frequency representation (TFR). As the acquired signal consists of many components, like the meshing and rotating components of the gear and bearing, the fault component may be masked by other unrelated components. The MTEWT is used to separate the fault feature from the masking components. A variety of experimental signals of the wind turbine planetary gearbox under nonstationary conditions have been analyzed to demonstrate the effectiveness and robustness of the proposed method. Results show that the proposed method is effective in diagnosing both gear and bearing faults.

## 1. Introduction

Wind energy is the fastest growing renewable energy source due to its cleanness and reproducibility [1]. However, wind turbines are subjected to dynamic excitations such as varying wind speeds, electricity grid events, or sea waves [2,3]. They are more prone to failure than in-house generation units. As wind turbines are usually located at remote land/water locations that may be difficult to access, their maintenance is difficult, expensive, and challenging. Therefore, fault diagnosis and condition monitoring of wind turbines are important for reducing downtime and economic loss [4,5,6,7]. Furthermore, fault diagnosis and detection in wind turbines can be accomplished by sensor-based methods [8,9,10] (also called signal-based methods) or analytical model-based methods [11,12,13,14].

A planetary gearbox is widely used in wind turbines to meet the requirements of smaller size or higher torque output. In a wind turbine, the planetary gearbox transfers the mechanical energy from the rotor hub with a low rotational speed to the generator with a high speed. The planetary gearbox malfunctions easily due to severe working conditions, such as wide-range speed fluctuation, time-varying load, extreme operational temperature, and humidity changes [10]. The downtime and repair costs significantly increase the operation cost of wind turbines. Hence, the fault diagnosis for planetary gearboxes in wind turbines is a vital research topic. Many researchers have made considerable contributions to planetary gearbox fault diagnosis recently. Some scholars [15,16,17,18,19] studied the effects of gear faults, manufacturing errors, and loading on the vibration responses through dynamics modeling and analysis. Lei [20] and Bartelmus [21,22] respectively proposed statistical indices for the condition monitoring of a planetary gearbox under constant and nonstationary operations. Feng [23,24,25] summarized the spectral characteristics of planetary gearbox vibration signals.

The industrial trend of wind energy leans towards multi-megawatt and large wind turbines, whose size has increased from 20 kW to 5 MW during the last two decades [26,27]. To capture wind energy efficiently, most large wind turbines operate at varying speeds [10]. Consequently, the signals collected from vibration sensors mounted in the wind turbine planetary gearbox are characterized as nonstationary features. Extracting the time-variant fault information from these signals is challenging for the fault diagnosis of a wind turbine planetary gearbox under nonstationary conditions. Time-frequency analysis methods can effectively reveal the time-variant features and have been commonly used to extract the fault information from nonstationary signals [28,29]. Tang [30,31,32] presented different methods based on wavelet transform for extracting transient features for diagnosing wind turbine gearbox faults. Liu [27] used local mean decomposition (LMD) to analyze the gearbox vibration signal and to identify the fault characteristic frequency of the gear. Feng [33] employed the demodulation analysis methods based on ensemble empirical mode decomposition (EEMD) and energy separation to diagnose the chipped tooth fault of the sun gear. However, the short time Fourier transform (STFT) and wavelet transform are limited by the Heisenberg uncertainty principle [34]. The best time localization and finest frequency resolution cannot be accomplished simultaneously. The empirical mode decomposition method (EMD) has four drawbacks, i.e., lack of mathematical theory, boundary effect, mode mixing, and stop criteria [35,36]. The LMD method is ineffective in dealing with the signals containing components whose sidebands are closely spaced.

Recently, Daubechies [37] proposed synchrosqueezing transform (SST) to reveal the complicated time-frequency features of nonstationary signals. SST has been successfully used in a variety of fields, such as medicine [38], geophysics [39,40,41], and rotating machinery [42,43]. SST is a special reassignment algorithm [44,45] and corresponds to a nonlinear operator that improves the time-frequency (TF) resolution. In SST, the time-frequency coefficients are reallocated only in the frequency axis to preserve the causality [46]. However, SST and most of its variations [47,48,49,50] could only sharpen the time-frequency representation (TFR) for signals with “slowly varying” instantaneous frequency (IF). A novel SST method, named high-order SST, was proposed by Pham et al. [51]. This method defines new synchrosqueezing operators based on high order amplitude and phase approximations and produces a highly concentrated TFR for both signals with fast varying IF and slowly varying IF.

In this study, a time-frequency method based on high-order synchrosqueezing transform and multi-taper empirical wavelet transform is proposed for the fault detection of wind turbine planetary gearboxes under nonstationary conditions. The high-order SST produces a highly concentrated TFR. The signals of a wind turbine exhibit typically time-varying features due to the complex operating conditions, such as speed fluctuation and load variation. The original signal is resampled with the shaft speed to avoid the speed fluctuation and spectrum smearing. Generally, a tachometer is required to obtain the instantaneous speed to sample signals equi-angularly. This requirement is barely satisfied in many applications due to limited space and cost [52]. The instantaneous rotating speed is estimated via the dynamic path optimization ridge detection (DPORD) algorithm [53] based on the TFR. Also, the estimation precision is guaranteed by high-quality time-frequency distribution calculated by the high-order SST. Planetary gearbox signals consist of many complex components. Unwanted gear (sun, planet, and ring) and/or bearing components may exist and easily mislead the fault diagnosis [54]. Multi-taper empirical wavelet transform (MTEWT) is employed to separate the fault features of the planetary gearbox from other irrelevant components. The multi-tapering approach can reduce the variance and improve the accuracy of the power spectrum estimation. A signal is decomposed into a set of principal modes by an adaptive filter bank constructed by the MTEWT. Both simulated and experimental vibration signals have verified the performance of the proposed method on fault diagnosis for planetary gearbox of wind turbines under nonstationary conditions.

The rest of this paper is organized as follows. Section 2 describes the proposed method in detail, especially the high-order SST algorithm and the MTEWT algorithm. Section 3 gives the procedure of the proposed method in detecting faults of the wind turbine planetary gearbox under nonstationary conditions. In Section 4, the effectiveness and robustness of the proposed method is verified by two experiments. Finally, conclusions are given in Section 5.

## 2. The Proposed Method

### 2.1. High-Order SST

As a post-processing method, SST has been used for other TFRs [42,49,55]. In this paper, the derivation process of the high-order SST is based on the STFT-based SST.

The signal acquired by the vibration sensor mounted in the wind turbine planetary gearbox can be modeled as a typical amplitude-modulated and frequency-modulated (AM-FM) signal. The high-order SST achieves a highly energy concentrated TFR for an AM-FM signal by using accurate approximations of the amplitude and phase.

An AM-FM signal is modeled as:

(1) $$s(\tau )=A(\tau )e^{i2\pi \phi (\tau )}$$

where *A*(*τ*) and *φ*(*τ*) are the amplitude and phase functions; both are real functions.

Then the STFT of this signal at time *t* and frequency *f* is defined as:

(2) $$V_{s}^{g}(t,f)=\int s(\tau )g^{*}(\tau -t)e^{-i2\pi f(\tau -t)}d\tau$$

where *g* is the window function and g* is the complex conjugate of *g*.

The traditional SST is defined as follows:

(3) $$T_{s}^{g,\varepsilon }(t,\omega )=\frac{1}{g^{*}(0)}\int_{\left\{|V_{s}^{g}(t,f)|>\varepsilon \right\}}V_{s}^{g}(t,f)\delta (\omega -{\hat{\omega }}_{s}(t,f))df$$

where *ε* is the threshold and *δ* is the Dirac distribution. ${\hat{\omega }}_{s}\left(t,f\right)$ denotes the IF and is estimated as:

(4) $${\hat{\omega }}_{s}(t,f)=R\{\frac{\partial_{t}V_{s}^{g}(t,f)}{i2\pi V_{s}^{g}(t,f)}\}$$

where *R*{*Z*} represents the real part of *Z* and ∂t is the partial derivative with respect to *t*.

In SST, the coefficients $V_{s}^{g}\left(t,f\right)$ are reassigned in the time-frequency plane. However, the SST method uses a zero-order estimation to extract the true IF. It only works well in processing a signal with a slowly time-varying IF. In the high-order SST method, the IF estimation is based on high order Taylor expansions of the amplitude and phase of a signal to improve the energy concentration of the TFR.

The Taylor expansion of the signal in Equation (1) for *τ* close to *t* is given as:

(5) $$s(\tau )=\exp(\sum_{k=0}^{N}\frac{{\left[\log(A)\right]}^{\left(k\right)}(t)+i2\pi \phi^{\left(k\right)}(t)}{k!}{\left(\tau -t\right)}^{k})$$

where *F*^(*k*)^(*t*) denotes the *k*th derivative of *F* at *t*.

Then Equation (2) is written as:

(6) $$\begin{array}{l} V_{s}^{g}(t,f)=\int s(\tau +t)g^{*}(\tau )e^{-i2\pi f\tau }d\tau \\ \;=\int \exp(\sum_{k=0}^{N}\frac{\left[\log(A){]}^{\left(k\right)}(t)+i2\pi \phi^{\left(k\right)}(t)\right)}{k!})\tau^{k}g^{*}(\tau )e^{-i2\pi f\tau }d\tau \end{array}$$

The STFT $V_{s}^{g}\left(t,f\right)$ is differentiated with respect to *t* and divided by $i2\pi V_{s}^{g}\left(t,f\right)$. The above equation is given as:

(7) $$\frac{\partial_{t}V_{s}^{g}(t,f)}{i2\pi V_{s}^{g}(t,f)}=\frac{\left[\log(A){]}^{\prime }(t\right)}{i2\pi }+\phi^{\prime }(t)+\sum_{k=2}^{N}\frac{\left[\log(A){]}^{\left(k\right)}(t)+i2\pi \phi^{\left(k\right)}(t\right)}{i2\pi (k-1)!}\frac{V_{s}^{t^{k-1}g}(t,f)}{V_{s}^{g}(t,f)}$$

where $V_{s}^{t^{k-1}g}\left(t,f\right)$ is the STFT of the signal *s*(*t*) computed with window *t^k^*^−1^*g*(*t*).

The *N*th-order complex IF ${\tilde{\omega }}_{s}^{\left[N\right]}\left(t,f\right)$ is defined as:

(8) $${\tilde{\omega }}_{s}^{\left[N\right]}(t,f)=\frac{\left[\log(A){]}^{\prime }(t\right)}{i2\pi }+\phi^{\prime }(t)$$

Its real part ${\hat{\omega }}_{s}^{\left[N\right]}\left(t,f\right)=R\left({\tilde{\omega }}_{s}^{\left[N\right]}\left(t,f\right)\right)$ is the desired IF ϕ′(t). Moreover, ${\tilde{\omega }}_{s}^{\left[N\right]}\left(t,f\right)$ can be calculated as:

(9) $${\tilde{\omega }}_{s}^{\left[N\right]}(t,f)=\frac{\partial_{t}V_{s}^{g}(t,f)}{i2\pi V_{s}^{g}(t,f)}-\sum_{k=2}^{N}\frac{\left[\log(A){]}^{\left(k\right)}(t)+i2\pi \phi^{\left(k\right)}(t\right)}{i2\pi (k-1)!}\frac{V_{s}^{t^{k-1}g}(t,f)}{V_{s}^{g}(t,f)}$$

The modulation operator ${\tilde{q}}^{\left[k,N\right]}$ is introduced and defined as:

(10) $${\tilde{q}}^{\left[k,N\right]}=\frac{\left[\log(A){]}^{\left(k\right)}(t)+i2\pi \phi^{\left(k\right)}(t\right)}{i2\pi (k-1)!}$$

Then the *N*th-order complex IF ${\tilde{\omega }}_{s}^{\left[N\right]}\left(t,f\right)$ is written as:

(11) $${\tilde{\omega }}_{s}^{\left[N\right]}(t,f)=\frac{\partial_{t}V_{s}^{g}(t,f)}{i2\pi V_{s}^{g}(t,f)}-\sum_{k=2}^{N}{\tilde{q}}^{\left[k,N\right]}\frac{V_{s}^{t^{k-1}g}(t,f)}{V_{s}^{g}(t,f)}$$

Furthermore, ${\tilde{q}}^{\left[k,N\right]}$ can be calculated as:

(12) $${\tilde{q}}^{\left[k,N\right]}=\left\{ \begin{array}{l} y_{N}(t,f)\;if\;k=N;\\ y_{k}(t,f)-\sum_{j=k+1}^{N}x_{j,k}(t,f){\tilde{q}}^{\left[j,N\right]}\;if\;k<N; \end{array}\right.$$

where $y_{j}\left(t,f\right)$ and $x_{k,j}\left(t,f\right)$ are defined as follows:

(13) $$y_{j}(t,f)=\left\{ \begin{array}{l} \frac{\partial_{t}V_{s}^{g}(t,f)}{i2\pi V_{s}^{g}(t,f)}\;if\;j=1;\\ \frac{\partial_{f}y_{j-1}(t,f)}{\partial_{f}x_{j,j-1}(t,f)}\;if\;j>1; \end{array}\right.$$

(14) $$x_{k,j}(t,f)=\left\{ \begin{array}{l} \frac{V_{s}^{t^{k-1}g}(t,f)}{V_{s}^{g}(t,f)}\;if\;j=1;\\ \frac{\partial_{f}x_{k,j-1}(t,f)}{\partial_{f}x_{j,j-1}(t,f)}\;if\;j>1; \end{array}\right.$$

Then the *N*th-order SST (SSTN) is defined by replacing ${\hat{\omega }}_{s}\left(t,f\right)$ by ${\hat{\omega }}_{s}^{\left[N\right]}\left(t,f\right)$ in Equation (3):

(15) $$T_{N,s}^{g,\varepsilon }(t,\omega )=\frac{1}{g^{*}(0)}\int_{\left\{|V_{s}^{g}(t,f)|>\varepsilon \right\}}V_{s}^{g}(t,f)\delta (\omega -{\hat{\omega }}_{s}^{\left[N\right]}(t,f))df$$

A simulated signal s(t)=A(t)cos(2πϕ(t)) is used to evaluate the performance of the high-order SST. The signal is sampled at 4096 Hz. Its amplitude and phase are given as:

(16) $$\left\{ \begin{array}{l} A(t)=10+20t^{2}\\ \phi (t)=1200t-5e^{-1.5t}\sin(30\pi t) \end{array}\right.$$

**Figure 1:**
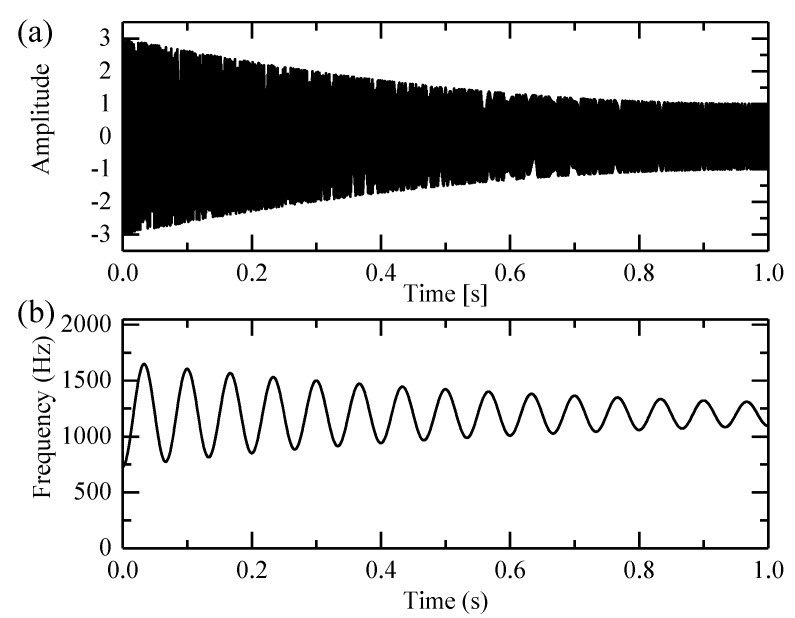
The simulated signal: (**a**) Its time domain waveform and (**b**) its instantaneous frequency.

The signal and its IF are shown in Figure 1. The IF ranges from 700 Hz to 1600 Hz and varies in one second. The large and fast frequency variations make time-frequency analysis difficult.

**Figure 2:**
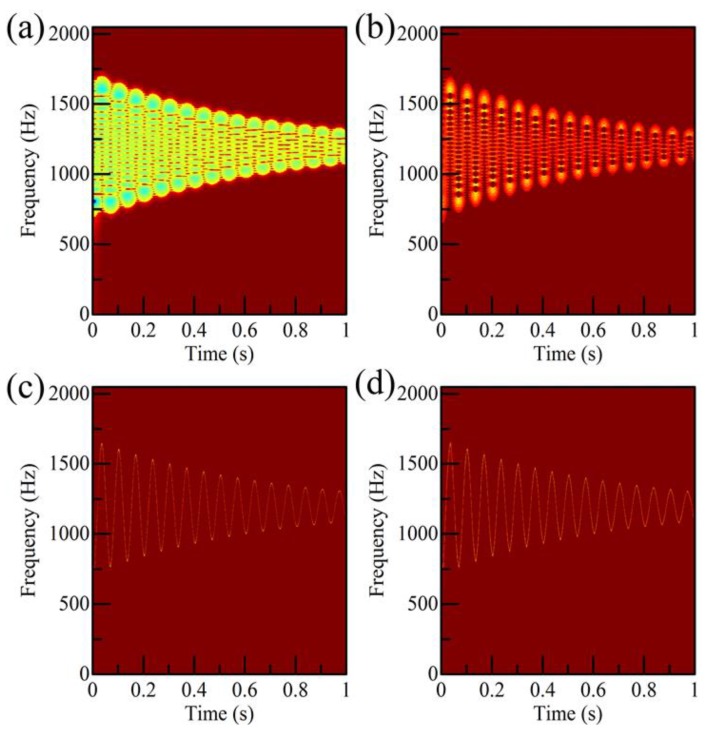
The time-frequency representation (TFR) provided by (**a**) STFT; (**b**) SST; (**c**) SST3; and (**d**) SST4.

The TFR provided by different time-frequency methods are compared in Figure 2. In the TFR provided by the STFT and the traditional SST methods, ridge smearing appears. The low time-frequency resolution will greatly decrease the precision of the IF estimation. On the other hand, the TFR provided by the third-order SST (SST3) and the forth-order SST (SST4) methods are much sharpener than those of the STFT and the traditional SST methods. The IF feature can be easily interpreted based on the sharpened TFR provided by the SST3 and SST4 approaches. Therefore, the high-order SST method can effectively improve the energy concentration of the TFR for the signal with large and fast frequency variations.

SST3 and SST4 seem to behave similarly in terms of the energy concentration of the TFR. Furthermore, computational costs of four time-frequency methods are considered. The algorithms are run on a computer with an Intel Core i5-4460 CPU and 8.0 GB RAM. Then results of the computational costs are listed in Table 1. The computational cost of the SST3 method is shorter than that of the SST4 method. For the sake of computational efficiency, the SST3 method is adopted in this study.

### 2.2. Multi-Taper Empirical Wavelet Transform

The multi-taper empirical wavelet transform algorithm is proposed to identify the fault features from a planetary gearbox vibration signal. As the original empirical wavelet transform (EWT) method yields unexpected segmentation due to false peaks and burrs induced by noisy and non-stationary factors, the multi-tapering is used to overcome these shortcomings. The multi-tapering approach can reduce the variance and improve the accuracy of the power spectrum estimation. In this approach, several orthonormal functions are used as tapers to estimate the corresponding power spectrum. The multi-taper spectral estimate is defined as:

(17) $$S^{mt}(f)=\frac{1}{L}\sum_{k=0}^{L-1}S_{k}^{mt}(f)$$

where

(18) $$S_{k}^{mt}(f)={\left|\sum h_{k}(n)x(n)e^{-i2\pi fn}\right|}^{2}$$

And *h_k_*(*n*) is the *k*th taper orthogonal to other tapers:

(19) $$\sum h_{k}(n)h_{j}(n)=\delta_{kj}$$

In the spectrum segmentation part, boundaries of each segment are determined by the spectrum peaks indicating dominant components. These peaks are picked out based on the spectrum shape. Then the boundary is described as the minimum in two consecutive frequency peaks. Details of the spectrum segmentation can be found in References [56,57].

Inspired by the construction of Littlewood-Paley and Meyer’s wavelets, the empirical wavelets are constructed as bandpass filters. The empirical scaling function and empirical wavelets are given by the following expressions:

(20) $$\Phi_{n}(\omega )=\left\{ \begin{array}{l} 1\;\mathrm{if}\;\left|\omega \right|\leq \omega_{n}-\tau_{n}\;\\ \cos\left[\frac{\pi }{2}\nu \left(\frac{1}{2\tau_{n}}(\left|\omega \right|-\omega_{n}+\tau_{n})\right)\right]\;\mathrm{if}\;\omega_{n}-\tau_{n}\leq \left|\omega \right|\leq \omega_{n}+\tau_{n}\;\\ 0\;\mathrm{otherwise} \end{array}\right.$$

(21) $$\Psi_{n}(\omega )=\left\{ \begin{array}{l} 1\;\mathrm{if}\;\omega_{n}+\tau_{n}\leq \left|\omega \right|\leq \omega_{n+1}-\tau_{n+1}\\ \cos\left[\frac{\pi }{2}v\left(\frac{1}{2\tau_{n+1}}(\left|\omega \right|-\omega_{n+1}+\tau_{n+1})\right)\right]\;\mathrm{if}\;\omega_{n+1}-\tau_{n+1}\leq \left|\omega \right|\leq \omega_{n+1}+\tau_{n+1}\\ \sin\left[\frac{\pi }{2}v\left(\frac{1}{2\tau_{n}}(\left|\omega \right|-\omega_{n}+\tau_{n})\right)\right]\;\mathrm{if}\;\omega_{n}-\tau_{n}\leq \left|\omega \right|\leq \omega_{n}+\tau_{n}\\ 0\;\mathrm{otherwise}. \end{array}\right.$$

To obtain a wavelet tight frame, the parameter *τ_n_* satisfies the following equation:

(22) $$\tau_{n}<\omega_{n}\min_{n}(\frac{\omega_{n+1}-\omega_{n}}{\omega_{n+1}+\omega_{n}})$$

According to the traditional wavelet theory, both approximated coefficients and detailed coefficients are given by the inner products:

(23) $$W_{f}^{\varepsilon }(0,t)=\langle f,\phi_{1}\rangle =\int f(\tau )\overset{─}{\phi_{1}(\tau -t)}\;d\tau$$

(24) $$W_{f}^{\varepsilon }(n,t)=\langle f,\psi_{n}\rangle =\int f(\tau )\overset{─}{\psi_{n}(\tau -t)}\;d\tau$$

Then the empirical modes are defined as:

(25) $$f_{0}(t)=W_{f}^{\varepsilon }(0,t)*\phi_{1}(t)$$

(26) $$f_{k}(t)=W_{f}^{\varepsilon }(k,t)*\psi_{k}(t)$$

An example is given to compare the performance of the EWT and the MTEWT method. The test signal is defined as:

(27) $$signal=\left\{ \begin{array}{l} \cos(1.5t)\cdot \sin(100\pi t)\;0<t\leq 0.5\\ 0.1\sin(400t)\;0.5<t\leq 1 \end{array}\right.$$

**Figure 3:**
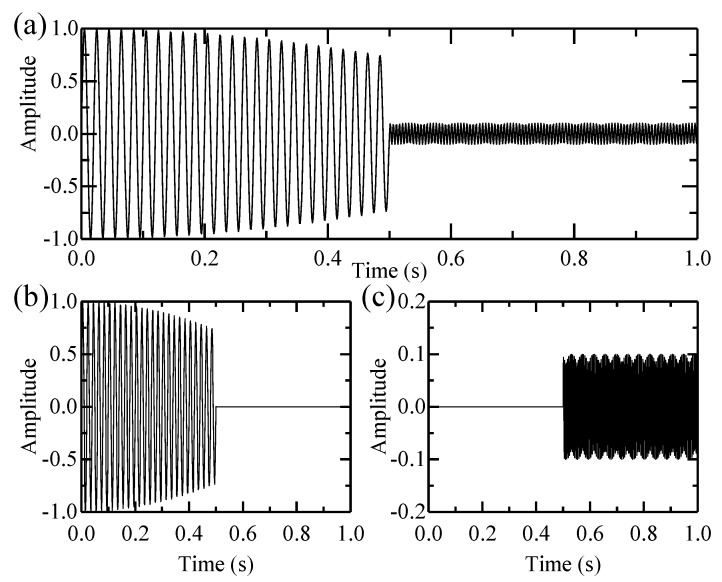
(**a**) The test signal; (**b**) its low-frequency component and (**c**) its high-frequency component.

**Figure 4:**
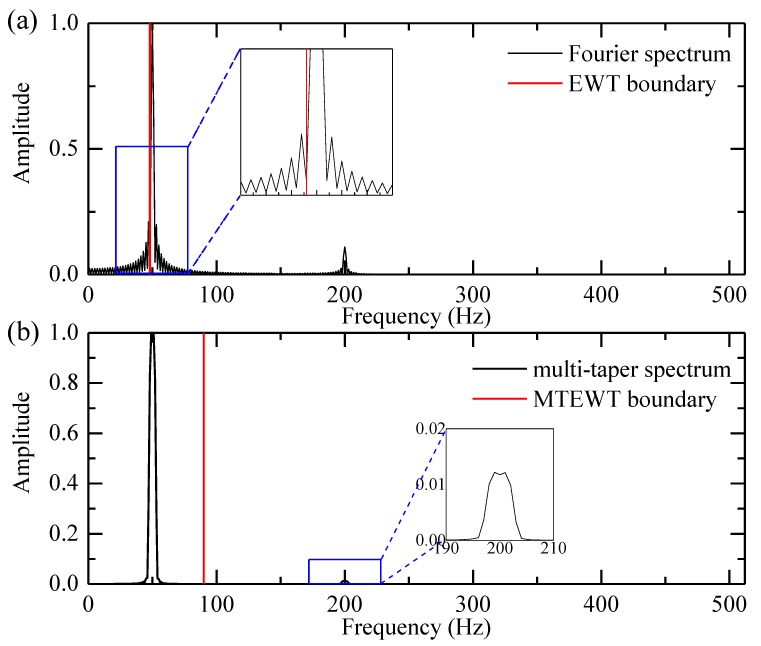
The EWT and MTEWT detected boundaries: (**a**) The Fourier spectrum and the EWT detected boundary; (**b**) the multi-taper spectrum and the MTEWT detected boundary.

**Figure 5:**
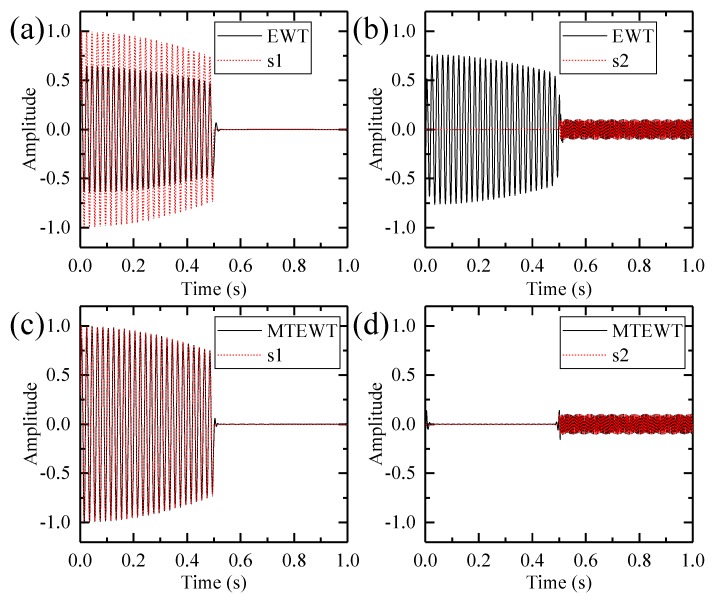
The EWT and MTEWT decomposition results: (**a**) The first EWT mode and the low-frequency component; (**b**) the second EWT mode and the high-frequency component; (**c**) the first MTEWT mode and the low-frequency component; (**d**) the second MTEWT mode and the high-frequency component.

This signal and its constituents are shown in Figure 3. The low-frequency and high-frequency components only exist in certain time periods. Then the EWT and MTEWT methods are used to decompose the signal. The Fourier and the multi-taper spectrum along with boundaries detected by both decomposition methods are depicted in Figure 4. The empirical modes decomposed by the EWT and MTEWT are shown in Figure 5.

In Figure 4a, some false peaks occur in the Fourier spectrum due to the data truncation or other nonstationary factors, which result in the improper boundary. In the EWT decomposition results shown in Figure 5a,b, the low-frequency component and the high-frequency component are mixed together.

In Figure 4b, the curve of the multi-taper spectrum is smooth and the boundary detected by the MTEWT is reasonable. In Figure 5c,d, both the low-frequency and high-frequency components are clearly recovered by the MTEWT methods. Therefore, the MTEWT method performs better than the EWT method in decomposing the nonstationary signal.

## 3. Procedure of the Proposed Method

To detect the faults of the wind turbine planetary gearbox under nonstationary conditions, a new time-frequency method based on high-order SST and MTEWT is proposed in this study. The fault features are separated from masking components or speed-related components. The procedure of the proposed method is summarized as follows:

(1) Obtain the time-frequency representation via the high-order SST

A sharpened TFR is obtained via the high-order SST. Compared with other time-frequency methods, the high-order SST provides a highly concentrated time-frequency representation, which guarantees the precision of IF estimation.

(2) Estimate the IF and resample the signal synchronous to the shaft instantaneous speed

The IF is a crucial feature to recognize the time-frequency pattern of nonstationary signals in the wind turbine. In the present study, the IF is estimated directly from the vibration signal, as the provision of a tachometer with sufficient resolution is not always possible due to technological or cost reasons. The IF is estimated based on the DPORD algorithm. Details about the DPORD algorithm can be found in References [53,58].

Under nonstationary conditions, the fault features of the vibration signal vary with time. Therefore, the direct application of spectrum-based methods leads to spectrum smearing and false diagnosis. The original signal is resampled at constant angular increments of the estimated shaft instantaneous speed. In this way, the spectrum smearing phenomenon is avoided and speed-related components are separated.

(3) Apply the MTEWT algorithm to extract the fault feature

The wind turbines exhibit complex dynamic responses due to extreme loading and widely varying operating conditions. The fault features are masked by unrelated components originating from other machine elements like shafts or gears. The resampled signal is decomposed by the MTEWT method and the fault features are separated from masking components.

## 4. Experiments

The effectiveness of the proposed method is validated by vibration signals collected from two experiments. The first experiment is carried out on a drivetrain dynamics simulator. The second experiment is performed on a wind turbine planetary gearbox of an actual wind turbine. Meanwhile, the proposed method is also compared with the fast kurtogram method to show its effectiveness.

### 4.1. Experiment 1: A Machinery Fault Simulator

**Figure 6:**
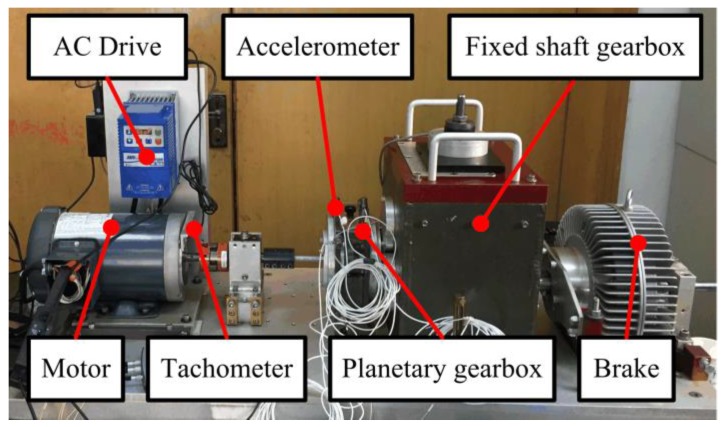
The test rig of the wind turbine planetary gearbox.

**Figure 7:**
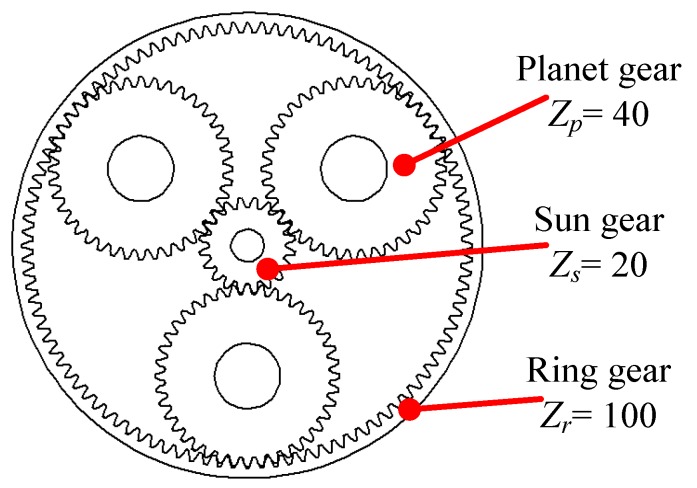
Schematic of the planetary gearbox.

The vibration data are collected from a Spectra Quest’s drivetrain dynamics simulator, which is used as a fault simulation of the wind turbine planetary gearbox. As shown in Figure 6, the simulator contains an alternating current (AC) motor, a planetary gearbox, a fixed shaft gearbox, and a magnetic brake. A tachometer and an accelerometer are used to collect the shaft speed signal and the gear vibration signal, respectively. The shaft rotating speed is manipulated by a speed controller. The sampling rate is 12,800 Hz, and the time length for the record is 20 s. The schematic view and gear parameters of the planetary gearbox are given in Figure 7.

**Figure 8:**
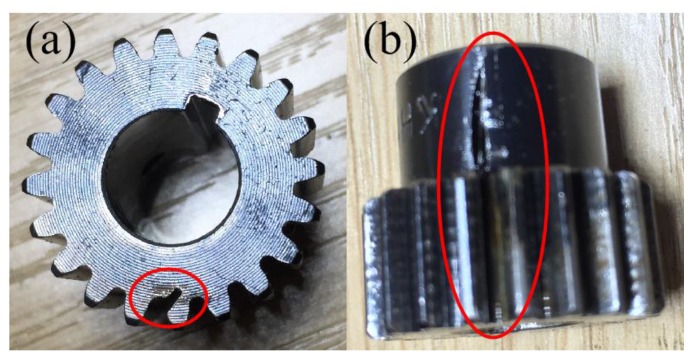
The faulty sun gear with tooth crack: (**a**) The top view and (**b**) the side view.

The effectiveness of the proposed method is verified by detecting the tooth crack in a sun gear which is given in Figure 8. The sun gear fault order with reference to the rotating sun gear is 2.5.

**Figure 9:**
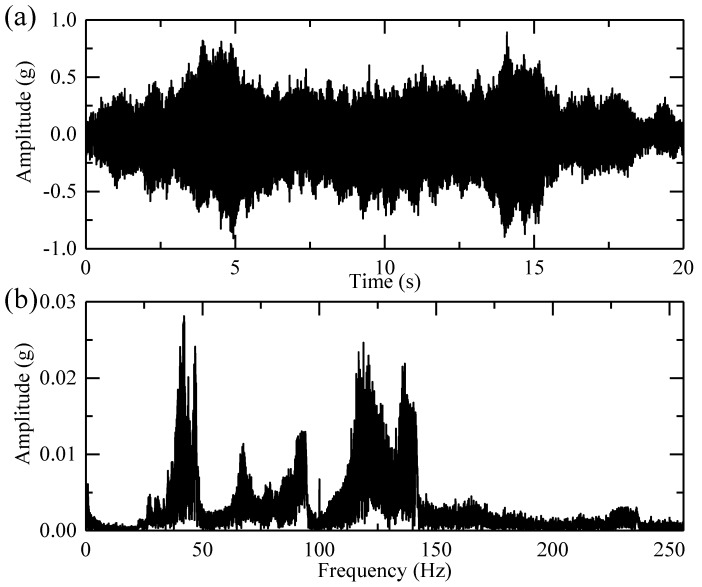
The sun gear fault signal: (**a**) Its time domain waveform and (**b**) its spectrum.

The collected vibration signal and its spectrum are depicted in Figure 9. Many peaks appear in the Fourier spectrum and it is difficult to associate these peaks with fault characteristic frequencies or orders due to the speed variation.

**Figure 10:**
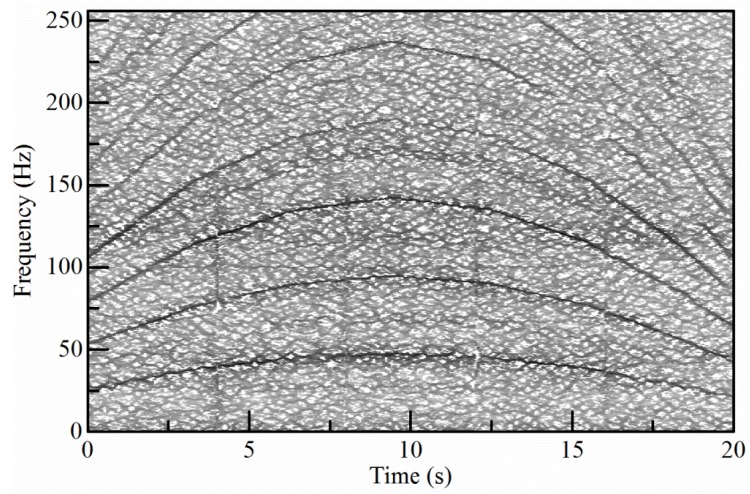
The TFR of the sun gear fault signal.

To reveal the crack fault, the proposed method is employed to analyze the vibration signal. Firstly, the TFR of the signal is calculated via the high-order SST and shown in Figure 10.

**Figure 11:**
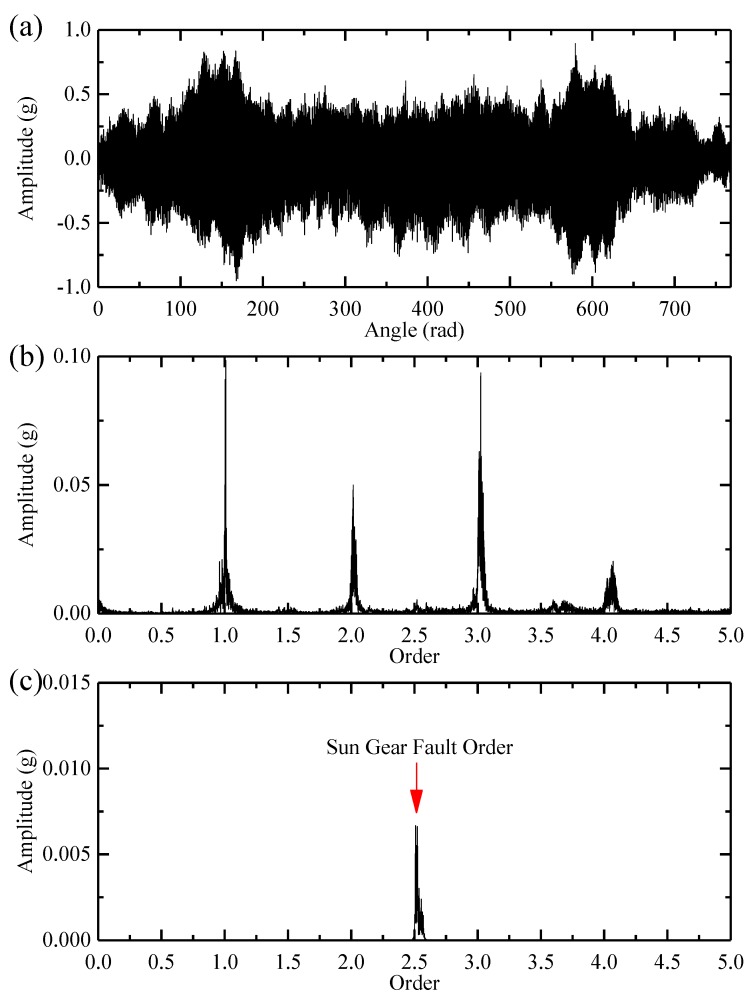
(**a**) The resampled sun gear signal; (**b**) the order spectrum of the resampled signal; and (**c**) the order spectrum of empirical mode 6.

Secondly, the IF is estimated via the DPORD method based on the calculated TFR. Thirdly, the original signal is resampled synchronous to the estimated IF. The resampled signal and its order spectrum are shown in Figure 11a,b. However, the order spectrum fails to provide sufficient information about the sun gear crack fault.

Finally, the resampled signal is decomposed into 13 empirical modes via the MTEWT method. As shown in Figure 11c, the sun gear fault order can be identified in the order spectrum of empirical mode 6. The identified fault order, marked with a red arrow, is 2.52, which is very close to the theoretical value of 2.50. Therefore, the sun gear fault can be effectively diagnosed.

### 4.2. Experiment 2: A Wind Turbine Planetary Gearbox

The second set of experimental data is collected from a wind turbine planetary gearbox and is provided by the diagnosis contest during the International Conference on Condition Monitoring of Machinery in Non-Stationary Operations (CMMNO) in 2014.

**Figure 12:**
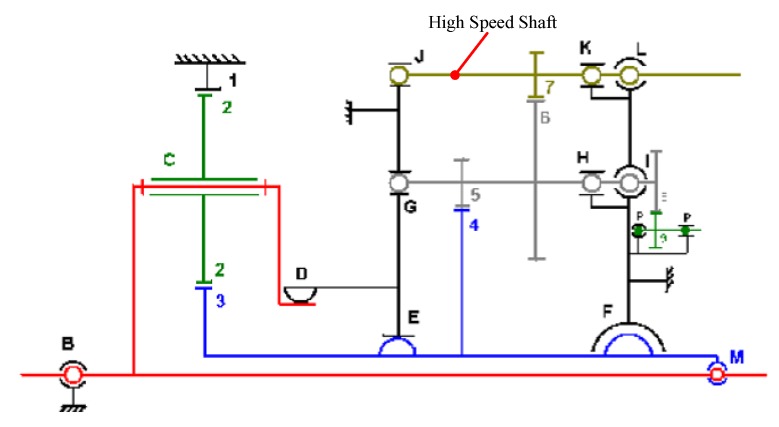
The planetary gearbox kinematics [59].

The vibration signal is measured on the gearbox casing of a wind turbine in the radial direction. The kinematics of the wind turbine planetary gearbox is described in Figure 12. The vibration signal is sampled at 5 kHz with a time length of approximately 550 s. The amplitude of this signal is normalized to unitary maximum amplitude. A tachometer is located on the high-speed shaft (carrying gear #7 in Figure 12) to give a reference speed signal. A fault is set on the inner race of the planet bearing and generates an angular periodic impulsive phenomenon at 0.191 high-speed shaft orders in the signal [59]. And parameters of the planetary gearbox are given in Table 2.

The first goal is to estimate the instantaneous angular speed of the high-speed shaft directly from the vibrational signal in order to address the case where no provision of a tachometer with sufficient resolution is made available for technological or cost reasons. The estimated result is compared with the reference speed signal. The second goal is to diagnose the bearing faults in the wind turbine planetary gearbox under nonstationary working conditions in the industrial field. In this realistic case, fault features do not correspond to priori expectations, leading to potential misinterpretation or false diagnosis.

**Figure 13:**
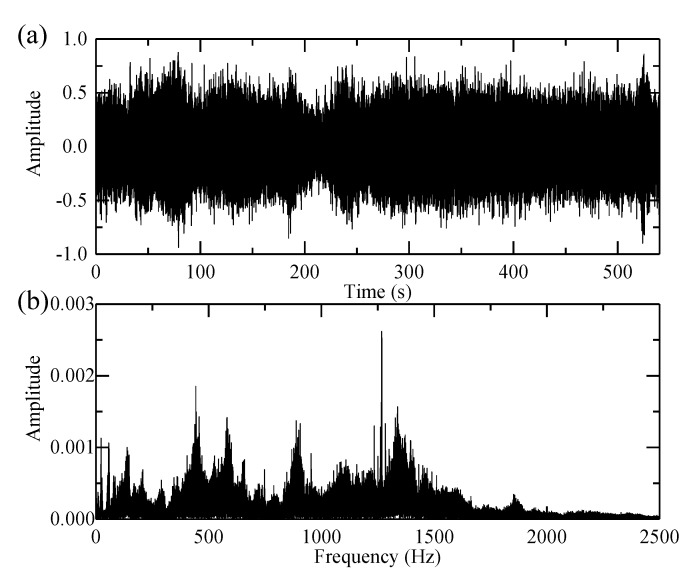
(**a**) The planet bearing fault signal provided by the Conference on Condition Monitoring of Machinery in Non-Stationary Operations (CMMNO) and (**b**) its envelope spectrum.

**Figure 14:**
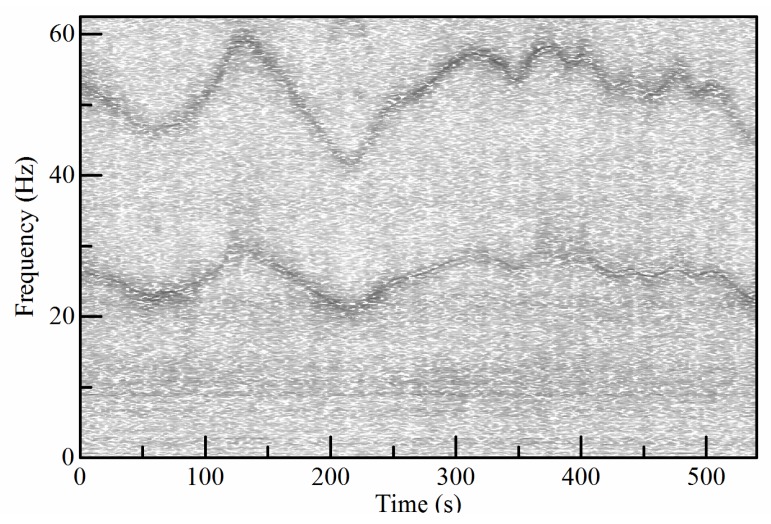
The TFR of the CMMNO vibration signal.

The vibration signal and its spectrum are given in Figure 13. The high-order SST algorithm is employed to give a sharpened TFR, which is shown in Figure 14a.

**Figure 15:**
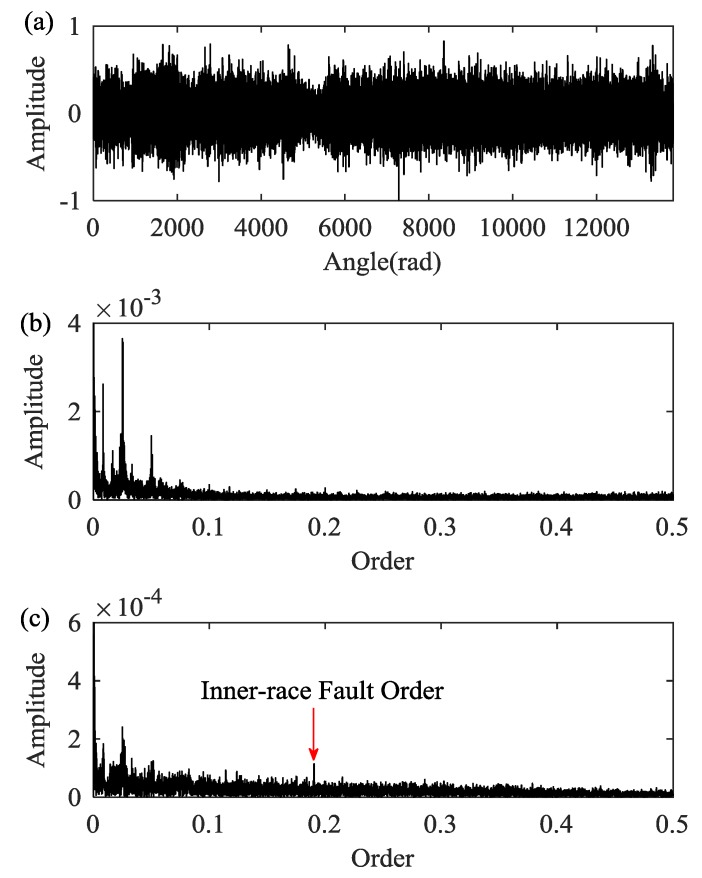
(**a**) The resampled signal; (**b**) the envelope order spectrum of the resampled signal; and (**c**) the envelope order spectrum of empirical mode 5.

Then the DPORD algorithm is employed to estimate the IF. Furthermore, the signal is resampled in an angular domain to avoid the influences of speed fluctuation. The resampled signal is shown along with its envelope order spectrum in Figure 15a,b. The resampled signal is decomposed into seven modes. As show in Figure 15c, the inner-race fault order can be identified in the envelope order spectrum of empirical mode 5. The detected inner-race fault order is 0.1903, which is very close to the theoretical value of 0.191. Therefore, the proposed method reveals the presence of the inner-race fault in one of the planet bearings.

**Figure 16:**
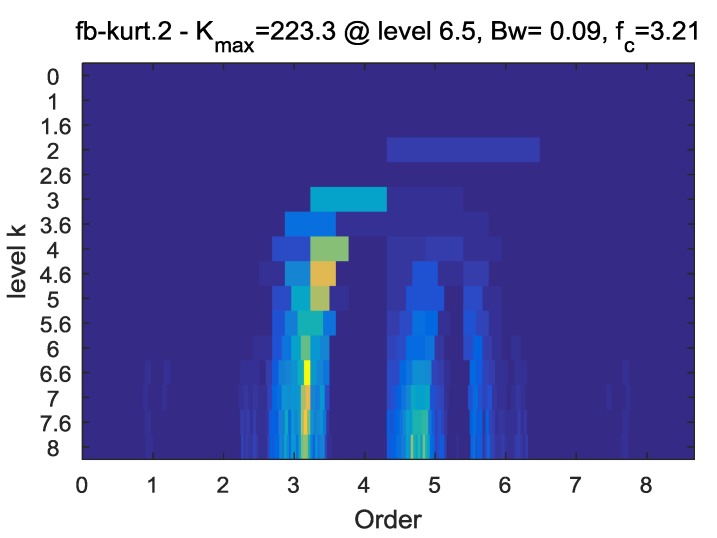
The kurtogram of the resampled signal.

**Figure 17:**
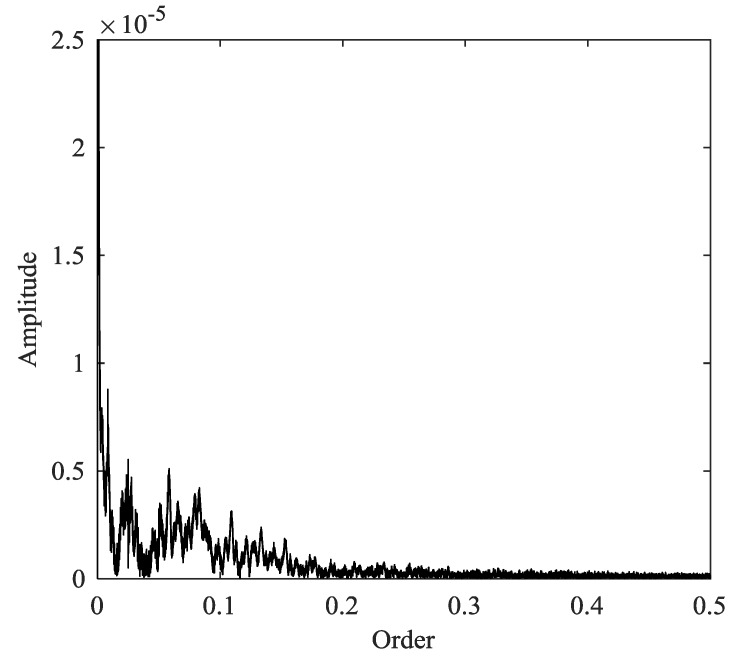
The envelope order spectrum of the filtered signal.

For comparison, the fast kurtogram method is also used to analyze the resampled signal to extract the fault feature. The kurtogram is shown in Figure 16 and an optimal filter with the center frequency of 3.21 and the bandwidth of 0.09 is determined. Then the resampled signal is filtered based on the above parameters and the envelope order spectrum of the filtered signal is shown in Figure 17. However, the inner-race fault order is indistinguishable in the envelope order spectrum of the filtered signal. The proposed method is more efficient than the fast kurtogram method in extracting the fault feature in this experiment.

## 5. Conclusions

In this study, a novel time-frequency method based on high-order SST and MTEWT is proposed for the fault diagnosis of a wind turbine planetary gearbox under nonstationary conditions. The high-order SST defines new synchrosqueezing operators based on high order amplitude and phase approximations. Compared with other time-frequency analysis methods, the high-order SST produces a highly concentrated TFR for both signals with fast varying IF and slowly varying IF. Meanwhile, the MTEWT method is proposed to separate the fault feature from masking components originating from other machine elements such as shafts or gears.

The effectiveness of the proposed method is validated by a variety of time-varying experimental signals measured by the vibration sensors mounted in the wind turbine planetary gearbox under nonstationary conditions. The calculated TFR is highly energy-concentrated and the estimated IF matches very well with the actual IF. The proposed method can successfully detect the sun gear crack fault, the planet gear crack fault, multi gear faults, and the planet bearing fault. Results indicate that the proposed method is a promising tool for the fault diagnosis and condition monitoring of a wind turbine planetary gearbox.

## Figures and Tables

**Table 1:** Computational costs of four time-frequency methods for a simulated signal.

Method	STFT	SST	SST3	SST4
Computational cost (s)	4.01	7.45	22.12	42.52

**Table 2:** Gear parameters of the planetary gearbox.

Gear	1	2	3	4	5	6	7	8	9
Gear teeth	123	50(3)	21	93	22	120	29	63	23
